# Rasch Modeling and Multilevel Confirmatory Factor Analysis for the Usability of the Impact of Event Scale-Revised (IES-R) during the COVID-19 Pandemic

**DOI:** 10.3390/healthcare10101858

**Published:** 2022-09-24

**Authors:** Musheer A. Aljaberi, Kuo-Hsin Lee, Naser A. Alareqe, Mousa A. Qasem, Abdulsamad Alsalahi, Atiyeh M. Abdallah, Sarah Noman, Ala’a B. Al-Tammemi, Mohamed Izham Mohamed Ibrahim, Chung-Ying Lin

**Affiliations:** 1 Faculty of Medicine and Health Sciences, Taiz University, Taiz 6803, Yemen; 2 Department of Community Health, Faculty of Medicine and Health Sciences, Universiti Putra Malaysia, Serdang 43300, Malaysia; 3 Faculty of Nursing and Applied Sciences, Lincoln University College, Petaling Jaya 47301, Malaysia; 4 Department of Emergency Medicine, E-Da Hospital, I-Shou University, No. 1, Yi-Da Road, Yanchao District, Kaohsiung City 824, Taiwan; 5 School of Medicine, I-Shou University, No. 8, Yi-Da Road, Jiao-Su Village, Yan-Chao District, Kaohsiung City 824, Taiwan; 6 Department of Educational Psychology, Faculty of Education, Taiz University, Taiz 6803, Yemen; 7 Department of Pharmaceutical Technology, Faculty of Pharmacy, University of Malaya, Kuala Lumpur 50603, Malaysia; 8 Department of Pharmacology, Faculty of Pharmacy, Sana’a University, Mazbah District, Sana’a 1247, Yemen; 9 Department of Biomedical Sciences, College of Health Sciences, QU-Health, Qatar University, Doha 2713, Qatar; 10 Migration Health Division, International Organization for Migration (IOM), Amman 11953, Jordan; 11 Department of Clinical Pharmacy and Practice, College of Pharmacy, QU-Health, Qatar University, Doha 2713, Qatar; 12 Institute of Allied Health Sciences, College of Medicine, National Cheng Kung University, Tainan 701, Taiwan

**Keywords:** COVID-19, post-traumatic stress disorder (PTSD), advanced psychometrics, Impact of Event Scale-Revised (IES-R), Rasch model, multilevel confirmatory factor analysis

## Abstract

Background: Several instruments are currently used to assess Coronavirus Disease 2019 (COVID-19) -induced psychological distress, including the 22-item Impact of Event Scale-Revised (IES-R). The IES-R is a self-administered scale used to assess post-traumatic stress disorder (PTSD). The current study aimed to examine the construct validity of the IES-R, based on the Rasch model, with COVID-19-related data, as well as to test the multilevel construct validity of the IES-R within and among countries during the pandemic crisis. Methods: A multi-country web-based cross-sectional survey was conducted utilizing the 22-item IES-R. A total of 1020 participants enrolled in our survey, of whom 999 were included in the analyses. Data were analyzed using Rasch modeling and multilevel confirmatory factor analysis (MCFA). Results: The Rasch modeling results of the IES-R demonstrated that the IES-R is a satisfactory instrument with the five-point Likert scale, asserting that its 22 items are significant contributors to assessing PTSD as a unidimensional construct covered by the items of the IES-R. The MCFA confirmed that the 22-item IES-R, with its three factors, including intrusion, avoidance, and hyperarousal, demonstrates adequate construct validity at the within- and among-country levels. However, the results of the Akaike information criterion (AIC) model determined that the 16-item IES-R is better than the 22-item IES-R. Conclusion: The results suggested that the 22-item IES-R is a reliable screening instrument for measuring PTSD related to the COVID-19 pandemic, and can be utilized to provide timely psychological health support, when needed, based on the screening results.

## 1. Introduction

Recently, the COVID-19 pandemic has spread as an evolving infectious disease that is mainly caused by novel strains of severe acute respiratory syndrome coronavirus (SARS-CoV-2) [1,2,3,4]. In December 2019, the first outbreak of the COVID-19 pandemic was detected in Wuhan, China, and then spread throughout the world, to be declared by the World Health Organization (WHO) as a Public Health Emergency of International Concern (PHEIC) and a pandemic on 30th January and 11th March 2020, respectively. The outbreak spread of the COVID-19 pandemic incidents showed fluctuating trends worldwide, both between waves and within a wave, between declining and rising. Despite the recent reports on the declining trend of this pandemic since the last peak in March 2022, there has been an increase in the number of weekly cases for the fifth consecutive week from the beginning of June 2022 to 13 July 2022, with over 5.7 million weekly new cases reported, and over 9800 deaths reported. During the week of 1–7 August 2022, the number of confirmed cases reported exceeded 6.9 million new weekly cases. Yet, the rate of new weekly cases appeared to decline, as it was estimated to be 24% during the period of 8 to 14 August 2022, with over 5.4 million new cases, when compared to the rate of cases reported during the previous week. By 5th September 2022, the number of confirmed cases reached 600,366,479, which was reported by the WHO. This includes a number of 6,460,493 fatalities across the globe. Therefore, it is clear from the statistics that although vaccinations and precautions have been taken over the world, the future course seems uncertain [3,4,5,6,7,8]. Pandemic-induced stress has resulted in significant socio-economic and psychological repercussions [9,10,11,12,13,14,15,16,17].

Additionally, the COVID-19 pandemic has induced many negative psychological responses, which significantly burden mental wellbeing [4,18,19,20,21,22]. The COVID-19-related news and rumors, conspiracy beliefs amongst the general public during the early stages of the crisis, the spread of rumors and fake information through mass media and social media, the lack of vaccines during the early pandemic period, and inequity in vaccine distribution or health care provision, as well as disease-associated stigma, were all reported in the literature to be associated with significant impacts on mental health [22,23,24,25,26,27,28,29,30]. Moreover, the COVID-19-induced emotional reactions due to infection and pandemic-associated mitigation measures (e.g., lockdown, facemasks, travel restrictions, etc.) have intensified mental symptomatology among different populations, regardless of their previous health status, as pandemic mitigation strategies and control measures varied between different countries in terms of timing, stringency, and duration. As the pandemic spread was faster than expected, the high possibility of contracting SARS-CoV-2, lack of definitive treatment and vaccines at the beginning of the crisis, post-COVID-19 pandemic sequelae, the possibility of re-infection even after being fully vaccinated, and pandemic fatigue are believed to be contributing factors and risks for suffering from mental health issues [20,21,29,31,32,33,34].

The rapid and extensive spread of the COVID-19 pandemic worldwide, besides the unprecedented changes in daily life, has left people alarmed and frightened [15,20,30,35,36,37,38]. Historically, there have been multiple outbreaks over the past years, such as the Severe Acute Respiratory Syndrome (SARS) epidemic, when moderate to severe post-traumatic stress symptoms were reported in the highly affected areas [39]. Additionally, during the swine flu (influenza A H1N1) outbreak, a study showed that 9.6% and 32.9% of the general population were either severely or moderately worried about the possibility of being infected, respectively [40]. The ebola virus disease, Middle East Respiratory Syndrome (MERS), and SARS epidemics all showed an impact on mental health, including depression, stress, anxiety, and even substance abuse [41].

For the current pandemic, the Impact of Event Scale-Revised (IES-R) is probably one of the most widely used self-report measures feasible to assess traumatic stress caused by the COVID-19 pandemic. The IES-R measure is an easily self-administered questionnaire to assess the symptoms of Post-Traumatic Stress Disorder (PTSD) following traumatic events [42,43]. Additionally, the IES-R has been used in previous outbreaks, such as SARS and Swine Flu, as well as the COVID-19 pandemic [5,44,45,46,47,48,49,50,51,52]. Concerning the utility of the IES-R in the COVID-19 pandemic, the scale can measure the extent to which the respondents are distressed by COVID-19-related symptoms based on the three subscales of intrusion, avoidance, and hyperarousal, as experienced in the past seven days. IES-R scores have been deemed reliable and valid in previous studies, with different populations, assessing the psychological impact of SARS and the COVID-19 pandemic [5,47,49,53,54,55,56,57,58,59,60].

Although the IES-R was first introduced in the 1990s by Weiss and Marmar [42], it surprisingly has generated little evaluation of the psychometric properties and construct validity of the scale, during either this pandemic or previous traumatic events [5,23,48,54,61]. However, the IES-R showed high internal consistency for the three subscales (intrusion, avoidance, and hyperarousal), and the test–retest correlation coefficients ranged from 0.51 to 0.94 among a traumatic sample. The principal component factor analysis, with varimax rotation, revealed a strong single factor accounting for 49% of the variance. This result may be due to the possibility that not all subjects were experiencing high or even medium symptom levels [42,43].

The IES-R has been translated into several languages [23,54,56,58,62,63,64], with diverse findings regarding its factor structure. Specifically, prior findings suggest changing the factor structure of the IES-R from a single-factor structure to a three-factor structure, with items ranging from 10 to 20. For example, Brunet, et al., [64] found that the French IES-R has good internal consistency and slightly acceptable convergent validity. Additionally, the factor structure of the IES-R, which explained 56% of the variance retained in seven items of the hyperarousal factor, six items of the avoidance factor, and six items of the intrusion factor among 223 non-traumatized females who had recently experienced a natural disaster. Meanwhile, the results of Creamer, et al., [48] showed poor model fit, and the results of CFA did not show support for a three-factor solution corresponding to the three subscales of intrusion, avoidance, and hyperarousal among two samples of male Vietnam veterans: one seeking treatment for PTSD and another community sample with varying levels of traumatic stress symptomatology. Additionally, the exploratory factor analysis suggested either a single factor or two factors, assessing intrusion/hyperarousal and avoidance, with no clear statistical advantage for either model [48]. Moreover, Norhayati and Aniza [63] showed that only ten items of the IES-R fit with the three-factor model and were acceptable for use in measuring PTSD, demonstrating acceptable factor loadings among women who underwent a caesarian section in postnatal wards. Furthermore, the results of Baumert, et al. [65], in a sample experiencing a life-threatening cardiac event, indicated high reliability for the intrusion and avoidance subscales, and weaker reliability for the hyperarousal subscale. Hence, the hyperarousal subscale validity was arguable, and needed to undergo further investigations in this area [65].

Given that there is no consensus regarding the factor structure of the IES-R, additional investigations are needed on this topic. However, there is one potential method to use another advanced psychometric method (i.e., the Rasch model) to assist the traditional CFA in analyzing the factor structure of the IES-R. The Rasch model has the strength of converting ordinal scales (e.g., the five-point Likert scale used in the IES-R) to continuous scales for psychometric testing, regarding if any item in an instrument fits with the underlying construct measured by the instrument. Moreover, the Rasch model identifies whether the ordinal scales follow a monotonic order (i.e., the probabilities of answering a more severe level of PTSD are lower than the probabilities of answering a less severe level of PTSD) [66,67,68,69,70,71,72]. As such, the Rasch model helps identify potential IES-R items that fit the construct of PTSD. After using the Rasch models, multilevel confirmatory factor analysis (MCFA) could be conducted to account for the hierarchical structure of nested data being collected, e.g., within-level (individual) and between-level (among countries/cultures). The multilevel approach empowers us to inquire about several news questions regarding variables at higher levels of aggregation, such as effects related to countries, culture, etc. [73,74]. The previous studies on the IES-R discovered a dearth of knowledge on the construct validity of the IES-R, based on the Rasch model. Additionally, previous literature focused only on single-factor analysis. Accordingly, our present study aims (i) to examine the construct validity of the IES-R, based on the Rasch model, to assess PTSD during the COVID-19 pandemic, and (ii) to test the multilevel construct validity of the IES-R to assess the PTSD during the COVID-19 pandemic within countries and among countries.

## 2. Materials and Methods

### 2.1. Research Design

The researchers conducted Rasch models and MCFA through instrumental designs [75,76]. The current study used a sample collected via an online survey. The 22 items of the IES-R (see Supplementary Materials, Table S1) were adopted within a multi-country web-based survey. The data were collected through an electronic survey distributed to the study sample in twenty countries (see Table 1) in the English language. We decided to conduct our study using online platforms, using a Google form, as face-to-face surveying was not feasible due to the lockdown and the COVID-19 pandemic, as well as to eliminate geographical boundaries. The study participants were recruited using social media: WhatsApp, Facebook, and email.

### 2.2. Instruments

The Impact of Event Scale-Revised (IES-R) has 22 items [42], five of which were added to the original Horowitz, Wilner, and Alvarez (IES-R) [77] to better capture PTSD according to the criteria of the American Psychiatric Association Diagnostic and Statistical Manual of 176 Mental Disorders (DSM). This 22-item scale is factorized of three dimensions, namely: intrusion with eight items, 1, 2, 3, 6, 9, 14, 16, and 20; avoidance with eight items, 5, 7, 8, 11, 12, 13, 17, and 22; and hyperarousal with six items, 4, 10, 15, 18, 19, and 21 (Supplementary Materials, Table S1). The IES-R is designed with five Item Response Anchors rated from 0 to 4, where 0 indicates *not at all*; 1 = *a little bit*; 2 = *moderately*; 3 = *quite a bit*; and 4 = *extremely*. Subsequently, scores on the 22-item IES-R range from zero to eighty-eight.

### 2.3. Study Setting, Sample Size, and Sampling

Participants from multiple countries received our survey through various online platforms. In the cover letter of our survey, information about the purpose of our study, eligibility criteria, and informed consent was provided to participants. The survey was entitled Psychological and Behavioral Responses to the COVID-19 pandemic. Interested participants could click on a link to be directed to our survey questionnaire, after consenting to participate. All procedures contributing to this work complied with ethical research standards. Informed consent was obtained from all participants, and our study was approved by the Taiz University Research Ethics Committee on 26 March 2020, with the reference number: Taiz/RSCGS/2020/03/26/0236.

Regarding within-level sample size, which refers to participants in each group or country, it varied from 21 to 282. Multilevel modeling can be used to account for the complex design of individuals nested or clustered within the country. The merit of cluster sampling contributes an additional source of variation for higher levels. Subsequently, cluster sampling is much more common than other types of sampling in the multilevel approach, where individuals are related to a higher level, such as a country [78,79].

A total of 1020 participants enrolled in our survey, of whom 21 did not provide their informed consent and therefore were excluded from our analyses. The remaining 999 eligible participants were from 20 countries (Table 1). The number of participating countries represented the minimum number required to conduct multilevel modeling (between-level); as reported by Selig, et al., [80], a sample size of 20 countries is the minimum number for multilevel modeling.

### 2.4. Study Participants

Alongside the 22-item IES-R, our survey questionnaire solicited socio-demographic information, including gender, marital status, educational level, and employment status. Table 2 represents the demographic profile of the participants. Female participants slightly predominated the overall sample (*n* = 554; 55.5%). In addition, 46.4% of participants were single, while 49.6% were married. Most participants were students (*n* = 551; 55.2%), followed by participants who work in an educational profession (*n* = 230; 23%). A total of 60.5% (*n* = 604) of participants held postgraduate degrees (Masters or PhDs).

### 2.5. Data Analyses

We conducted descriptive statistics, Rasch measurement modeling, and MCFA, which is a type of structural equation model that is based on the analysis of covariance structure [81,82,83]. The detailed steps of these analyses are described in the following sections.

#### 2.5.1. Descriptive Statistics, Reliabilities, and Correlation

Descriptive statistics and cross-tabulation for a demographical variable were performed in IBM SPSS, Version 25 (IBM, Armonk and North Castle, NY, USA). Descriptive statistics for items included mean and standard deviation for each item and each dimension of the IES-R. Skewness (≤−/+3) and kurtosis (≤−/+7) were used as indicators for normal distribution [83]. Squared multiple correlation (SMC ≥ 0.30) and McDonald’s ω for each dimension of the IES-R, and the entire IES-R (≥0.70), are valuable requirements for subsequent advanced analysis. The McDonald’s ω, a modern style of reliability for psychological and psychiatry instruments [84,85,86,87], was performed in the current study in the JASP program.

#### 2.5.2. Rasch Measurement Modeling

To check the construct validity of the 22 items, Rasch measurement modeling (using Winsteps) was conducted to (i) test the validity of the five-point Likert scale of the Impact of Event scale, (ii) test fit statistics information for each item, as well as separation (≥2) and reliability (≥0.80) for both items and persons, and (iii) test the uni-dimensionality of the 22 items as a single dimension (e.g., event, in this context) based on an unexplained variance in first contrast with less than three items. Wright’s Map was presented to evaluate the interactive theoretical foundation of the 22 items and the participants [88,89]. Moreover, infit and outfit mean square were used to examine if any IES-R item had a misfit. A value between 0.5 and 1.5 for both infit and outfit mean square indicated that the item did not have a misfit [69].

#### 2.5.3. Bentler & Liang’s Maximum Likelihood Estimation Method

MCFA was conducted in EQS software, version 6.0 (IBM, Encino, Los Angeles, California, USA), based on Bentler & Liang’s maximum likelihood estimation method [90]. The merit of this estimation method applies to any sample size with a balanced or unbalanced design. It is most appropriate when the sample sizes vary substantially among clusters [90]. Previous studies [82,91,92] presented applicable studies on Bentler & Liang’s maximum likelihood estimation method. In our MCFA, a three-factor structure of the IES-R was examined. However, a misfit in the MCFA was remedied through the removal of items with low or nonsignificant factor loadings, to improve the data–model fit, until a satisfactory fit was achieved for the three-factor structure of the IES-R. The detailed procedures of this method involve five steps, as the following:

- Step 1: Perform a Single Conventional Confirmatory Factor Analysis

The objective of this step is to obtain goodness of fit statistics, which include the *p*-value of the Chi-Square statistic (non-significant), the Bentler–Bonett normed fit index (NFI) (≥0.90), the Bentler–Bonett non-normed fit index (NNFI) (≥0.90), the comparative fit index (CFI) (≥0.90), Bollen’s incremental fit index (IFI) (≥0.90), the Joreskog–Sorbom goodness of fit index (GFI) (≥0.90), the root mean square residual (RMR) (≤0.08), the standardized root mean square residual (SRMR) (≤0.08), the root mean square error of approximation (RMSEA) (≤0.08), and the 90% confidence interval of RMSEA (90% CI RMSEA) (≤0.08) [66,68,72,82,83,90,91,93,94,95]. The researcher can check for the re-specified model if the original model is a misfit until the researcher holds the fit model.

- Step 2: Estimation of Between-Group Variation

The intraclass correlation (ICC) of each item was invoked and created by the multilevel approach analyses. The ICC provides a descriptive tool for the proportion of country-level variation in each item of the IES-R. It ranges between 0.0 and 1. An ICC value close to zero shows that variation is at the individual-level model, whereas an ICC value close to 1.00 indicates the variation is at the country-level model [73,91,92].

- Step 3: Obtain Fitness of MCFA

The objective of this step is identifying and meeting the goodness of fit statistics (e.g., NFI (≥0.90), NNFI (≥0.90), CFI (≥0.90), IFI (≥0.90), GFI (≥0.90), RMR (≤0.08), SRMR (≤0.08), RMSEA (≤0.08), and 90% CI RMSEA (LO and UP) (≤0.08)) [66,67,72,82,91,92,96].

- Step 4: Estimate Within-Level Model

In this step, we have to check for statistically significant individual variations in each item with its analogous factors. Non-significant, low loading, or negative items do not contribute much to their respective dimension.

- Step 5: Estimate Between-Level Model

The objective of this step is to check for statistically significant country variations in each item with its equivalent factors. Non-significant, low loading, or negative items do not contribute much to their corresponding dimension. The statistical significance of the country-level loadings provides evidence about the extent to which the IES-R items are operatively effective in discriminating among countries.

The goodness of fit statistics, including Chi-Square (χ^2^), CFI, and RMSEA, were used to test the equivalence between the free/unconstrained MCFA model and constrained MCFA model [83,97]. Full equivalence occurs when the free/unconstrained MCFA model and the constrained MCFA model are equal in terms of goodness of fit statistics, as well as factor loadings between the two models, i.e., within-level and between-level. Partial equivalence occurs when the two models express equal goodness of fit statistics, along with variations in factor loadings.

Wald tests were conducted for factor loadings in the between-level model (country), whether there is a significant difference from zero, and the item’s ability to discriminate among countries’ levels. Wald tests are conducted by dividing each factor-loading by the parameter estimate’s standard error (S.E) [87]. Since this test follows a z-distribution, a value more than 1.96 is judged statistically significant at <0.05. The presence of both statistical significance and positive directions of the factor loading suggest that the IES-R may be effective in discriminating between the country level, notwithstanding its ability to distinguish between participants.

## 3. Results

### 3.1. Descriptive Statistics

Table 3 presents the descriptive parameters for the 22 items of the three dimensions of the IES-R: intrusion, avoidance, and hyperarousal factors. Squared multiple correlation (≥0.30) for each item in each dimension reaches standard criteria, which means that each item is related to the corresponding dimension by a given proportion. McDonald’s ω for each dimension exceeded the given criteria (≥0.70). Overall reliability for intrusion, avoidance, and hyperarousal exceeded the satisfactory standard coefficients (0.882\0.883, 0.862\0.864, and 0.832\0.837).

### 3.2. Rasch Measurement Model

**Figure 1:**
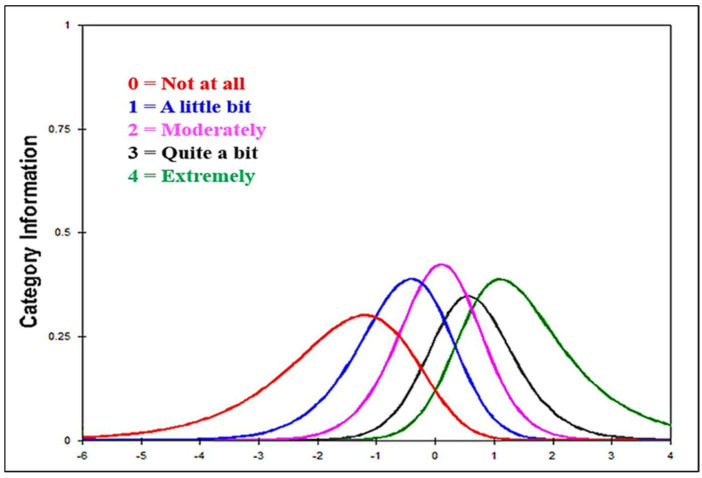
Probability model for the five Likert scales in the Impact of Event Scale-Revised.

The observed count for each Likert scale of impact for the IES-R exceeded the optimal number (20), varying from 10,174 (46%) for Likert *0* (*Not at all*) to 921 (4%) for Likert *4* (*extremely*). Moreover, the infit and outfit mean square for each Likert were clustered around 1. The category measure of the five Likert scales in the IES-R demonstrated a monotonic function starting from 2.38 for Likert *0* (*Not at all*) to 2.21 for *4* (*extremely*). The probability model (Figure 1) of the five Likert scales in the IES-R refers to no disordered Likert, meaning that the five Likert scales are appropriate to be used in the IES-R.

The point measure of correlation of the 22 items reached the acceptable standard (≥0.30), ranging from 0.44 (Q20_INT) to 0.64 (Q3_INT) (Table 4). The item-reliability of the IES-R was 0.98. The item-separation of the IES-R was 7. Similarly, the person-reliability of the IES-R was 0.88, and the person-separation was 2.

### 3.3. Wright’s Map of IES-R

**Figure 2:**
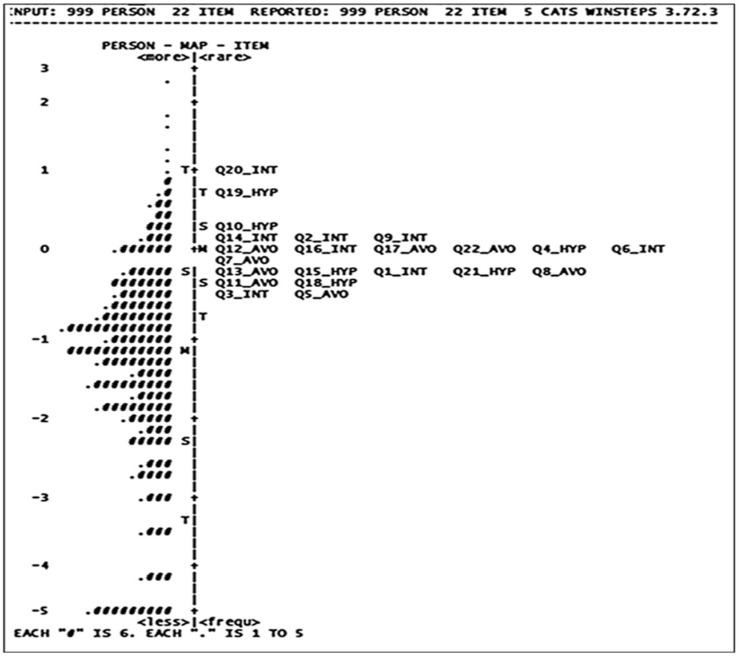
Wright map of the Impact of Event Scale-Revised (IES-R).

Table 4 shows the hierarchical orders of items based on the logits of each item, with its SE, from the highest difficulty item (Q20_Int = 1.05) to the lowest difficulty item (Q5_Avo = −0.47). The Wright map shown in Figure 2 is a graphical representation of the distribution of interaction between the 22 items of the IES, from easy items (e.g., Q3_INT and Q5_AVO), located at the bottom of the Wright map, to hard items (e.g., Q20_INT and Q19_HYP), situated on top of the map. The twenty two items of the IES-R were normally distributed between −2 and 2 values. The majority of participants were located between −2 and 2 values, as standard criteria.

### 3.4. Multilevel Confirmatory Factor Analysis for the IES-R

#### 3.4.1. Step 1: Performing Conventional CFA for the Total Sample Covariance Matrix

Conventional CFA of the 22-item IES-R failed to obtain acceptable standard of fit statistics (Table 5). A re-specified model was then examined by removing four items (Q3_Int, Q14_Int, Q20_Int, and Q15_Hyp; Supplementary Materials, Table S1). Conventional CFA of this 18-item IES-R (Supplementary Materials, Figure S1) obtained the acceptable standard of fit statistics, with items having satisfactory loadings (Supplementary Materials, Table S2).

#### 3.4.2. Step 2: Estimation of Between-Level Variations

Estimated ICCs were conducted for the 22-item IES-R. Results of the ICCs indicated that sixteen items were ≥ 0.02, which were returned for further analysis. Therefore, six items with negligible ICC (Q1_INT, Q2_INT, Q7_AVO, Q13_AVO, Q18_HYP, and Q15_HYP) were removed. Values of ICCs across the sixteen items ranged from 0.015–0.072.

#### 3.4.3. Step 3: Fitness of Multilevel CFA for the IES-R 

**Figure 3:**
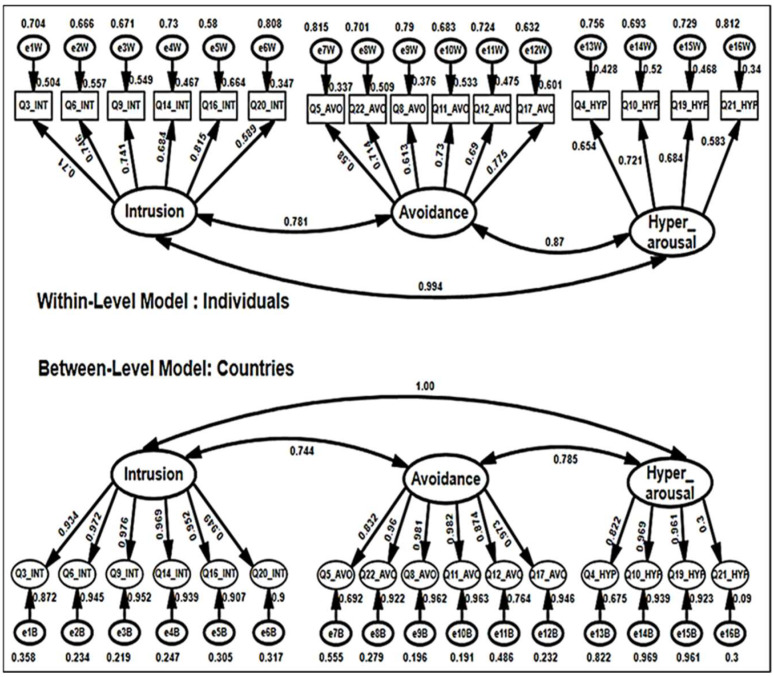
Multilevel confirmatory factor analysis for Impact of Event Scale-Revised. Intrusion (Int.), Avoidance (Avo.), and Hyperarousal (Hyp.).

MCFA of the 16-items IES-R converged for an admissible solution for the two levels, within and between. The goodness of fit statistics obtained plausible fit, as shown in Table 5. It can be concluded that within-level (individuals in each country) results of the IES-R scale are equivalent, invariant, or not differ from between-level (among countries) results, as pictured in Figure 3. Three latent constructs, intrusion with six items, avoidance with six items, and hyperarousal with four items, can be used with both within- and between-levels.

#### 3.4.4. Step 4: Estimation of Within-Level Model

Inter-correlation among three latent constructs of the within-level IES-R, intrusion, avoidance, and hyperarousal (*r* = 0.781 to 0.870), were all significant. Results of the parameters of multilevel analysis are presented in Table 6. The within-level analysis demonstrated that the six items in the intrusion dimension, six items in the avoidance dimension, and four items in the hyperarousal dimension were statistically significant, with a *z*-value above 1.964. That means that all items significantly contribute to their dimensions, indicating that all items contribute to the explanation and construction of their corresponding dimensions. 

#### 3.4.5. Step 5: Estimation of Between-Level Models

Between-level results of the multilevel analysis showed that six items in the intrusion dimension, six items in the avoidance dimension, and three out of four items in the hyperarousal dimension were statistically significant, with a *z*-value above 1.964 as the critical value (Table 6). The R-square value of the between-level model was of a substantial magnitude.

### 3.5. Examining for Measurement Invariance across Levels in the Analysis

The constrained multilevel model of all factor loadings within the individual model (within-levels) (*n* = 999) and among-countries model (between-levels) (*n* = 20) was performed simultaneously to assess whether a between-level model is equivalent to a within-level model. The Chi-Square difference in Table 5 = 47.629, indicates that the constrained model is not significantly worse at level 0.001, demonstrating an equivalence (no difference) between the two models, within-level vs. between-level.

The multilevel model analysis for this constrained IES-R produced fit statistics, including χ^2^, CFI, and RMSEA, which were then examined against free (unconstrained) multilevel model values for statistically significant differences. The equivalence procedure across the constrained and unconstrained multilevel models resulted in a statistically insignificant difference, χ2 = 34.528, at a significance level of 0.001. Moreover, differences in practical CFI and RMEAS between the two models were less than 0.01 and 0.015, respectively, which means that the difference in χ^2^ values, practical CFI, and RMEAS between the within- and between-level models did not establish poor fit results. 

Results of the between-levels model’s factor loadings were higher than their counterparts in the within-level model, which asserted the significance of the partial equivalence in the multilevel model. Notably, the proportion of unexplained variance was lower in the between-level model than in the within-level model, confirming that the between-country model is better than the within-individual model. In brief, the within- and between-levels models have partial equivalence, not full equivalence.

Wald Tests were applied to the within- and between-level models. Almost all factor loadings in the within-level model were statistically significant and positive, indicating that all items have the ability to distinguish participants with different levels of PTSD. Except for one item (Q21_HYP), all factor loadings were statistically significant in the between-level model, demonstrating that all items can differentiate between one country and another.

## 4. Discussion

The COVID-19 pandemic is a contagious illness caused by infection with SARS-CoV-2 [1,2,3,4]. From December 2019 to 5th September 2022, a total of 600,366,479 have been confirmed to be infected with the COVID-19 pandemic; among them, 6,460,493 died, as reported by the WHO [8]. Despite vaccination, further course of the COVID-19 pandemic is uncertain, and pandemic-induced stress has resulted in significant psychological repercussions [9,10,11,12,13,14,15,16]. In the literature, a number of studies tried to measure such post-traumatic psychological repercussions using the IES-R, a self-administered scale to assess PTSD after a traumatic event through measuring the symptoms of intrusion, avoidance, and hyperarousal [5,47,49,53,54,55,56,57,58]. Accordingly, this study used the Rasch analysis to examine the construct validity of the IES-R within the COVID-19 pandemic. The second objective aimed to test the multilevel construct validity of the IES-R.

Results from previous studies indicated that IES-R scores have been shown to be reliable and valid, with satisfactory internal consistency values in different populations, in assessing the psychological impact of different distress/traumatic events, such as SARS and, currently, the COVID-19 pandemic [5,47,49,53,54,55,56,57,58,59,60]. Similarly, the current study’s findings confirmed that the IES-R demonstrated a normal distribution and good reliability. Skewness and kurtosis indicators suggested that the 22 items of the IES-R were normally distributed. On the other hand, McDonald’s ω reliability confirmed that the 22 items of the IES-R obtained a high degree of reliability in both individual items and overall factors. Briefly, the initial basis of psychometric information is very well provided.

Additionally, the current findings indicate that the criteria of the five Likert scales in the IES-R (e.g., observed count, infit and outfit mean square, category measure, and probability model) met the Likert scale criteria of the Rasch model very well. The probabilities of choosing a *not at all* response in those without PTSD are much higher than those of choosing *a little bit*, *moderately*, *quite a bit*, or *extremely* responses. In other words, Rasch’s modeling results showed that the five-point scale corresponds well to the severity of PTSD. Moreover, both infit and outfit mean squares for all items were located within an acceptable range (≥0.60–≤1.60). That means that data on all items of the IES-R fit the Rasch model. The twenty two items of the IES-R are significant predictors and contributors in the approach to the IES-R being validated, suggesting theoretical and empirical fitness between data collected and the hypothesized concept of the IES-R, which Weiss and Marmar [42] assumed. Furthermore, the proportion of explained and unexplained variance supported the idea that the IES-R assesses a single construct (uni-dimension), and the second dimension is absent. Moreover, both person and item reliability in the IES-R was obtained at a high rate, suggesting that the IES-R is highly replicable within the population. Finally, a Wright map of the IES-R asserted positive interactions between items of the IES-R and participants who score those items. Items of the IES-R were able to classify the participants adequately, and vice versa. That is, people with different levels of PTSD can be distinguished using the IES-R items. Consistent with the findings of previous studies [42,43,48,59,63], this study confirms the construct validity of the IES-R in a single dimensionality, general distress, with three sub-factors and the ability differentiate between individuals with and without PTSD.

Results of ICC indicated that the twenty two items of the IES-R vary from 0.001 to 0.06, suggesting that sixteen items should be retained for multilevel constructs. A three-factor model of the 16-item IES-R, intrusion with six items, avoidance with six items, and hyperarousal with four items, fitted the within-level (within countries) and between-level (among countries) data. Partial invariances were found between the within-level and between-level. The three-factor model of the IES-R with 16 items fit both within-level and between-level. However, it was a better fit for the between-level (among countries) than within-level (within countries) model. Taken together, these results extend psychometric support for the IES-R from a multilevel perspective. The three-factor structure was supported in this investigation, consistent with previous research [59,60,63,64], with a difference in the number of items. Unlike the findings of Creamer et al. [48], this suggests that the IES-R is not simply a measure of general distress. Differences in methodology may account for the discrepancy between these studies. For example, Creamer et al. [48] used markedly different recruitment strategies to enroll clinical and nonclinical participants, possibly resulting in heterogeneity within the total sample. This heterogeneity conceivably could explain the single-factor solution noted by these authors, which might reflect general distress. Finally, results indicated that the three-factor model of the 16-item IES-R is a scale with soundly good psychometrics that can be used to scale the COVID-19 pandemic either within a country/culture or among countries. Psychological distress in the community is prevalent during the COVID-19 pandemic [20]. Thus, screening for psychological-related outcomes and planning preventive measures is paramount in maintaining an individual’s psychological wellbeing.

Theoretically, as well as based on the revealed statistics, this study’s findings confirm the validity and reliability of the IES-R as a satisfactory diagnostic tool used to measure the psychological impact of the COVID-19 pandemic, both at the within-country level and among different populations in different countries. Additionally, the current study findings could be reproduced by applying the IES-R measure in future studies. Practically, large-scale outbreaks and global pandemics impose a significant burden on the psychological wellbeing of humans. Therefore, and given the COVID-19 pandemic-associated health, socio-economic, and political impacts, in addition to the insufficient mental health policies in many countries, the findings of our study suggest the diagnostic utility of the IES-R to screen for PTSD and the psychological impact of the COVID-19 pandemic. Furthermore, early detection of individuals who suffer from PTSD due to a traumatic event or are at higher risk of developing PTSD will greatly help in providing sufficient support, timely referrals to specialist care, and proper interventions to prevent further worsening of their clinical status and mental health. In addition, using a validated and reliable tool, such as the IES-R, for early screening of stress symptomology will help in bridging the gap between physical and mental health, thus improving quality of life and health outcomes among individuals with PTSD. Furthermore, the findings of this study could be used as a guide for further investigations on effectively assessing PTSD after a traumatic event, which could increase clinicians’ awareness of psychological outcomes after traumatic events.

### Strengths and Limitations 

This innovative study was the first to test the IES-R from the point of Rasch modeling’s confirmatory criteria. Moreover, this novel study was the first to test the IES-R using MCFA at the within-country and between-country levels, concluding that the IES-R is a reasonable instrument to be used in assessing the psychological effect of the COVID-19 pandemic. On the other hand, there are three popular approaches to conducting multilevel analysis: hierarchical linear modeling, multilevel modeling, and maximum likelihood estimation method. This study focused on a single approach of multilevel modeling called Bentler & Liang’s maximum likelihood estimation method.

The present study has the following limitations. First, the studied sample was from a general population. Although we did not examine exposure to COVID-19 infection among the respondents, vicarious trauma may exist through exposure to COVID-19 news and false information circulated on social media (e.g., death rates, the idea that the virus causing the disease was man-made as a biological weapon to target specific populations, etc.) [22,24,30]. Therefore, no accurate data are available on the participants’ physical or mental health status. Given that the IES-R is designed for people with clinical suspicion of PTSD, using the IES-R for the general population may cause some issues with probability plots and their transitions in Rasch modeling. Although the present study’s sample might encounter events associated with PTSD (e.g., being infected by COVID-19 or experiencing infected loved ones), we cannot ensure that all the participants had such PTSD experiences. Moreover, different countries experienced different levels of COVID-19 pandemic severity, and the post-traumatic experiences might vary substantially across countries. Furthermore, the IES-R was distributed to the study participants in English. Therefore, addressing a measure in English to respondents whose native tongue is not English (e.g., Arab, Afghan, Chinese, etc.), despite their level of English proficiency, may entail a limitation due to the interpretation of idioms, apart from cultural issues, which may affect the way in which they respond to symptom scales; for example, Arabs and Asians tend to report physical symptoms on a mental health scale, while Western people describe emotions more [17,21,29]. Second, we did not collect data on potential risk factors for PTSD. The study was also possibly affected by social desirability bias, in which respondents tend to respond favorably, thereby distorting responses. Third, we removed several items in the IES-R to achieve a better fit for the present sample. However, the removal of these items might be due to the fact that the present sample is different from other studies testing the psychometric properties of the IES-R. In other words, additional studies are needed to examine the IES-R factor structure in different populations, including clinical patients and community samples. Following the aforementioned limitation, one should know that different factor structures (e.g., four-factor and five-factor) have been proposed for the IES-R [23]. Therefore, the removal of seven items in our study, to make the IES-R fit a three-factor structure, may cast doubt on the purity of its three-dimensional model. In this regard, further examination of alternative models for the IES-R is warranted, especially considering self-destructive/reckless behavior, as well as negative alterations in cognitions and mood symptoms into consideration [19,74].

## 5. Conclusions

The current study aimed to examine the construct validity of the IES-R, based on the Rasch model, in the COVID-19 pandemic, as well as test the multilevel construct validity of the IES-R in the COVID-19 pandemic both within and among countries. The Rasch model’s confirmatory construct validity criteria for the IES-R showed the adequacy of the five-point response categories of the scale for assessing PTSD as a unidimensional construct, covered by the items of the IESR. MCFA established that the IES-R with three factors, intrusion, avoidance, and hyperarousal, is supported at the within- and between-country levels. However, the results of the AIC model in CFA indicate that the 16-item IES-R is better than the 22-item IES-R. The findings suggested that the 22-item IES-R is a reliable screening tool for measuring PTSD related to the global pandemic of COVID-19, and can be utilized to provide timely psychological health support, when needed, based on the screening results. In terms of the theoretical and practical implications, the IES-R can be a satisfactory diagnostic tool used to measure PTSD and the psychological impact of the COVID-19 pandemic. At the same time, the findings of this study could be reproduced and implemented in MCFA at the within- and between-country levels in future studies. Moreover, using a validated and reliable tool, such as IES-R, for the early detection and diagnosis of stress symptomology will help in improving quality of life and health outcomes among individuals with PTSD, as well as supporting proper interventions to prevent further worsening clinical and mental health status.

## Figures and Tables

**Table 1:** List of countries and sample size.

Countries	Sample Size	Percentage
Malaysia	282	28.2
Yemen	111	11.1
Indonesia	73	7.3
Nigeria	62	6.2
Sri Lanka	65	6.5
Tunisia	43	4.3
Pakistan	27	2.7
Somalia	24	2.4
Syria	22	2.2
Saudi Arabia	22	2.2
Vietnam	23	2.3
Bangladesh	29	2.9
China	23	2.3
India	20	2.0
Iraq	34	3.4
Egypt	22	2.2
Algeria	24	2.4
Guinea	21	2.1
Afghanistan	25	2.5
Others	47	4.7
Total	999	100.0

**Table 2:** Socio-demographic profile of the participants.

	*n*	%
**Gender**		
Female	554	55.5
Male	445	44.5
**Marital status**		
Single	464	46.4
Married	496	49.6
Engaged	27	2.7
Divorced	12	1.2
**Employment status**		
Students	551	55.2
Healthcare workers	53	5.3
Educational profession	230	23.0
Administrative professional	56	5.6
Other	109	10.9
**Education level**		
High school equivalent	31	3.1
Bachelor	285	28.5
Diploma	79	7.9
Master	367	36.7
PhD	237	23.7

**Table 3:** Descriptive statistics, normality, and internal consistency of the items of the Impact of Event Scale-Revised (IES-R).

Items	Mean ± SD	Skewness (≤−/+3)	Kurtosis (≤−/+7)	Corrected Item Total Correlation	Squared Multiple Correlation (≥0.30)	McDonald’s ω (≥0.70)
Q1_Int	1.09 ± 1.048	0.872	0.208	0.610	0.424	0.873
Q2_Int	0.87 ± 1.116	1.249	0.750	0.568	0.337	0.877
Q3_Int	1.23 ± 1.099	0.746	−0.159	0.714	0.559	0.862
Q6_Int	0.98 ± 1.072	0.991	0.304	0.703	0.515	0.863
Q9_Int	0.86 ± 1.079	1.194	0.664	0.684	0.506	0.865
Q14_Int	0.86 ± 1.043	1.136	0.608	0.615	0.418	0.872
Q16_Int	0.91 ± 1.059	1.093	0.551	0.757	0.593	0.857
Q20_Int	0.41 ± 0.890	2.364	5.056	0.528	0.293	0.880
Overall Intrusion	7.21 ± 6.23					0.883
Q5_Avo	1.29 ± 1.249	0.707	−0.521	0.534	0.292	0.856
Q7_Avo	0.93 ± 1.149	1.165	0.492	0.493	0.251	0.860
Q22_Avo	0.94 ± 1.128	1.102	0.414	0.661	0.474	0.842
Q8_Avo	0.99 ± 1.179	1.054	0.175	0.608	0.383	0.849
Q11_Avo	1.20 ± 1.189	0.780	−0.304	0.712	0.527	0.836
Q12_Avo	0.99 ± 1.070	0.973	0.286	0.612	0.402	0.848
Q13_Avo	1.03 ± 1.077	0.895	0.128	0.554	0.336	0.854
Q17_Avo	0.98 ± 1.174	1.070	0.217	0.709	0.527	0.836
Overall Avoidance	8.35 ± 6.58					0.864
Q4_Hyp	0.97 ± 1.080	1.014	0.268	0.666	0.470	0.799
Q18_Hyp	1.11 ± 1.213	.913	−0.147	0.701	0.527	0.792
Q10_Hyp	0.79 ± 1.034	1.231	0.753	0.600	0.385	0.814
Q15_Hyp	1.04 ± 1.241	1.051	0.029	0.601	0.413	0.812
Q19_Hyp	0.55 ± 0.961	1.831	2.701	0.599	0.385	0.815
Q21_Hyp	1.04 ± 1.226	1.027	0.064	0.486	0.251	0.833
Overall Hyperarousal	5.50 ± 5.00					0.837
IES-R Overall	21.06 ± 16.30					

Note: Intrusion (Int.), Avoidance (Avo.), and Hyperarousal (Hyp.), S.D. = Standard deviation.

**Table 4:** Rasch parameters of the Impact of Event Scale-Revised (IES-R).

Items	Point Measure Correlation (≥0.03)	Infit Mean Squares (≥0.60–≤1.60)	Outfit Mean Squares (≥0.60–≤1.60)	S.E.	Logits Scores	Ordered Rank
Q1_Int	0.56	0.97	1.27	0.04	−0.21	18
Q2_Int	0.55	1.13	1.10	0.04	0.10	6
Q3_Int	0.64	0.78	0.86	0.04	−0.40	21
Q6_Int	0.63	0.78	0.82	0.04	−0.05	11
Q9_Int	0.57	0.97	0.89	0.04	0.12	5
Q14_Int	0.60	0.84	0.84	0.04	0.13	4
Q16_Int	0.64	0.71	0.71	0.04	0.04	7
Q20_Int	0.44	1.45	1.04	0.05	1.05	1
Q5_Avo	0.58	1.23	1.33	0.04	−0.47	22
Q7_Avo	0.49	1.41	1.43	0.04	0.02	8
Q22_Avo	0.57	1.06	1.05	0.04	0.00	9
Q8_Avo	0.53	1.30	1.41	0.04	−0.08	14
Q11_Avo	0.60	1.03	1.07	0.04	−0.36	20
Q12_Avo	0.63	0.78	0.82	0.04	−0.07	13
Q13_Avo	0.58	1.00	1.05	0.04	−0.13	15
Q17_Avo	0.61	0.96	0.86	0.04	−0.06	12
Q4_Hyp	0.61	0.85	0.88	0.04	−0.04	10
Q18_Hyp	0.63	0.93	0.92	0.04	−0.24	19
Q10_Hyp	0.60	0.85	0.76	0.04	0.24	3
Q15_Hyp	0.56	1.25	1.32	0.04	−0.14	16
Q19_Hyp	0.53	1.06	0.80	0.05	0.69	2
Q21_Hyp	0.54	1.31	1.40	0.04	−0.15	17

Note: Intrusion (Int.), Avoidance (Avo.), and Hyperarousal (Hyp.), S.E. = Standard error, Raw variance explained by measures = 45.0% (Modeled), Unexplained variance in 1st contrast = (2.5) 6.3% (Modeled), Separation and Reliability for Items = 7 and 0.98, Separation and Reliability for Person = 2 and 0.88.

**Table 5:** Goodness of fit indices for Impact of Events Scale-Revised (IES-R); single-level and two-level confirmatory factor analysis (CFA).

Indices	Acceptable Criteria	Conventional CFA		Multilevel CFA	Multilevel CFA		Constraint MCFA	Differences
		Original Model (22 Items)	Re-Specified Model (18 Items) *	Re-Specified Model (16 Items) **	Original Model (22 Items)	Re-Specified Model (16 Items) ***		
χ^2^	-	1940.189	911.344	943.261	1944.818	889.038	936.667	47.629
DF	-	206	132	264	412	202	215	13
*p*	>0.05	<0.001	<0.001	<0.001	<0.001	<0.001	<0.001	<0.001
NFI		0.839	0.900	0.902	0.846	0.900	0.900	0.000
NNFI	≥0.90	0.835	0.900	0.918	0.860	0.902	0.908	0.006
CFI	≥0.90	0.853	0.911	0.930	0.876	0.917	0.917	0.000
IFI	≥0.90	0.854	0.911	0.930	0.876	0.917	0.918	0.001
GFI	≥0.90	0.842	0.900	0.923	0.867	0.918	0.917	0.001
RMR	≤0.08	0.071	0.059	0.039	0.047	0.044	0.044	0.00
SRMR	≤0.08	0.058	0.048	0.048	0.052	0.047	0.079	0.032
RMSEA	≤0.08	0.092	0.077	0.067	0.083	0.080	0.078	0.002
90% CI RMSEA	≤0.08	(0.088–0.096)	(0.072- 0.082)	(0.062–0.072)	(0.080–0.087	0.075–0.086	0.073–0.084	0.002–0.002,
Model AIC	Less	1528.189	647.344	371.878	1075.934	485.038	506.667	

Note: χ^2^ = Chi-Square, DF = Degrees of freedom, *p* = Probability value for the Chi-Square statistic, NFI = Normed fit index, NNFI = Non-Normed fit index, CFI = Comparative fit index, IFI = Incremental fit index, GFI = Goodness of fit index, RMR = Root mean square residual, SRMR = Standardized root mean square residual, RMSEA = Root mean square error of approximation, 90% CI RMSEA = 90% Confidence interval of RMSEA, AIC = Akaike information criterion, CFA = Confirmatory factor analysis, MCFA = Multilevel confirmatory factor analysis, * 4 removed Items are Q3_Int, Q14_Int, Q20_Int, and Q15_Hyp ** 2 removed Items are Q1_INT and Q13_AVO, *** 6 removed Items are Q1_INT, Q2_INT, Q7_AVO, Q13_AVO, Q15_HYP, and Q18_HYP.

**Table 6:** Multi-level analysis: within-level model and between-level model.

Items	β		S.E		*z*-Value *		Loading		R. Square		PVUNE		Wald Tests	
	Within	Between	Within	Between	Within	Between	Within	Between	Within	Between	Within	Between	Within	Between
Intrusion														
Q3_INT	0.710	0.934	-	-	-	-	0.710	0.934	0.504	0.872	0.704	0.358	-	-
Q6_INT	0.746	0.972	0.046	0.216	22.486	4.950	0.746	0.972	0.557	0.945	0.666	0.234	16.22	4.50
Q9_INT	0.741	0.976	0.045	0.263	22.334	5.159	0.741	0.976	0.549	0.952	0.671	0.219	16.47	3.71
Q14_INT	0.684	0.969	0.044	0.189	20.638	4.307	0.684	0.969	0.467	0.939	0.730	0.247	15.55	5.13
Q16_INT	0.815	0.952	0.045	0.184	24.466	4.604	0.815	0.952	0.664	0.907	0.580	0.305	18.11	5.17
Q20_INT	0.589	0.949	0.038	0.166	17.808	4.950	0.589	0.949	0.347	0.900	0.808	0.317	15.5	5.72
Avoidance														
Q5_AVO	0.580	0.832	-	-	-	-	0.580	0.832	0.337	0.692	0.815	0.555	-	-
Q22_AVO	0.714	0.960	0.065	0.304	17.127	3.627	0.714	0.960	0.509	0.922	0.701	0.279	10.98	3.16
Q8_AVO	0.613	0.981	0.063	0.433	15.461	3.743	0.613	0.981	0.376	0.962	0.790	0.196	9.73	2.27
Q11_AVO	0.730	0.982	0.069	0.344	17.382	3.785	0.730	0.982	0.533	0.963	0.683	0.191	10.58	2.85
Q12_AVO	0.690	0.874	0.061	0.312	16.746	3.395	0.690	0.874	0.475	0.764	0.724	0.486	11.31	2.80
Q17_AVO	0.775	0.973	0.068	0.420	18.022	3.893	0.775	0.973	0.601	0.946	0.632	0.232	11.4	2.32
Hyperarousal														
Q4_HYP	0.654	0.822	-	-	-	-	0.654	0.822	0.428	0.675	0.756	0.822	-	-
Q10_HYP	0.721	0.969	0.051	0.378	20.372	3.554	0.721	0.969	0.520	939	0.693	0.969	14.14	2.56
Q19_HYP	0.684	0.961	0.047	0.443	19.472	3.548	0.684	0.961	0.468	0.923	0.729	0.961	14.55	2.17
Q21_HYP	0.583	0.300	0.060	0.371	16.915	0.969	0.583	0.300	0.340	0.090	0.812	0.300	9.72	0.81

Note: β = standardizing estimate (aka factor loading),— = path fixed for 1, *z*-value ≥ 1.964 refers to significant *p*-Value, S. E = Standard Error, PVUNE = Proportion of Variance Unexplained, Intrusion (Int.), Avoidance (Avo.), and Hyperarousal (Hyp.)

## Data Availability

The dataset that supports the findings of this study is not openly available and will be available from the corresponding author upon reasonable request.

## Supplementary Materials

The following supporting information can be downloaded at: https://www.mdpi.com/article/10.3390/healthcare10101858/s1, Figure S1: Confirmatory Factor Analysis for the IES-R; Table S1: Items of the IES-R; Table S2: Parameters of conventional confirmatory factor analysis for the Impact of Events Scale-Revised (IES-R).

## Abbreviations

AIC	Akaike information criterion
CFA	Confirmatory factor analysis
CFI	Comparative Fit Index
GFI	Goodness of Fit Index
ICC	Intraclass correlation
IES-R	Impact of Event Scale-Revised
IFI	Incremental fit index
MCFA	Multilevel confirmatory factor analysis
NFI	Normed Fit Index
NNFI	(Non) Normed Fit Index
PVUNE	Proportion of Variance Unexplained
PTSD	Post-traumatic stress disorder
RMSEA	Root mean square error of approximation
RMR	Root mean square residual
SRMR	Standardized root mean square residual
