# Supplementary material for: Rasch Modeling and Multilevel Confirmatory Factor Analysis for the Usability of the Impact of Event Scale-Revised (IES-R) during the COVID-19 Pandemic
Source: Healthcare (Basel). 2022 Sep 24;10(10):1858. doi: 10.3390/healthcare10101858 (PMC9602035; doi:10.3390/healthcare10101858)
Supplement: Supplementary file 1 [file healthcare-10-01858-s001.zip › healthcare-1900135-supplementary.pdf]

Supplementary Materials

Table S1. Items the Impact of Events Scale-Revised (IES-R)

Symbols	Items \Statement\ Questions
Q1_Int	Any reminder brought back feelings about it.
Q2_Int	I had trouble staying asleep.
Q3_Int	Other things kept making me think about it.
Q4_Hyp	I felt irritable and angry.
Q5_Avo	I avoided letting myself get upset when I thought about it or was reminded of it.
Q6_Int	I thought about it when I didn't mean to.
Q7_Avo	I felt as if it hadn't happened or wasn't real.
Q8_Avo	I stayed away from reminders of it.
Q9_Int	Pictures about it popped into my mind.
Q10_Hyp	I was jumpy and easily startled.
Q11_Avo	I tried not to think about it.
Q12_Avo	I was aware that I still had a lot of feelings about it, but I didn't deal with them.
Q13_Avo	My feelings about it were kind of numb.
Q14_Int	I found myself acting or feeling like I was back at that time.
Q15_Hyp	I had trouble falling asleep.
Q16_Int	I had waves of strong feelings about it.
Q17_Avo	I tried to remove it from my memory.
Q18_Hyp	I had trouble concentrating.
Q19_Hyp	Reminders of it caused me to have physical reactions, such as sweating, trouble breathing, nausea, or a pounding heart.
Q20_Int	I had dreams about it.
Q21_Hyp	I felt watchful and on-guard.
Q22_Avo	I tried not to talk about it.

Int =Intrusion, Avo =Avoidance, Hyp =Hyperarousal subscales

Table S2. Parameters of conventional confirmatory factor analysis for the Impact of Events Scale-Revised (IES-R)

	**β**	**S. E**	**T-value**	**Loading**	**R^2**	**PVUNE**	**Wald tests**	**ICC**
Intrusion								
Q1_INT	0.627	-	-	0.627	0.393	0.779	-	0.006
Q2_INT	0.633	0.063	17.101	0.633	0.401	0.774	10.05	0.019
Q6_INT	0.753	0.063	19.567	0.753	0.567	0.658	11.95	0.037
Q9_INT	0.731	0.063	19.128	0.731	0.534	0.683	11.60	0.069
Q16_INT	0.802	0.063	20.474	0.802	0.643	0.597	12.73	0.025
Avoidance								
Q5_AVO	0.586	-	-	0.586	0.344	0.810	-	0.034
Q7_AVO	0.533	0.059	14.152	0.533	0.284	0.846	9.03	0.026
Q22_AVO	0.721	0.063	17.623	0.721	0.520	0.693	11.44	0.042
Q8_AVO	0.642	0.064	16.272	0.642	0.413	0.766	10.03	0.076
Q11_AVO	0.744	0.067	17.986	0.744	0.554	0.668	11.10	0.046
Q12_AVO	0.700	0.059	17.275	0.700	0.490	0.714	11.86	0.045
Q13_AVO	0.611	0.057	15.696	0.611	0.373	0.792	10.72	0.001
Q17_AVO	0.779	0.067	18.503	0.779	0.606	0.627	11.63	0.091
Hyperarousal								
Q4_HYP	0.693	-	-	0.693	0.481	0.721	-	0.027
Q18_HYP	0.699	0.054	20.801	0.699	0.488	0.715	12.94	0.022
Q10_HYP	0.730	0.046	21.693	0.730	0.534	0.683	15.87	0.035
Q19_HYP	0.703	0.043	20.927	0.703	0.495	0.711	16.35	0.065
Q21_HYP	0.575	0.055	17.246	0.575	0.330	0.818	10.45	0.025
INT-AVO	0.772	0.041	10.578	0.772				
INT-HYP	0.982	0.055	9.709	0.982				
AVO-HYP	0.877	0.046	12.174	0.877				

β= standardizing estimate (aka factor loading), S.E =Standard Error, R^2= R square, PVUNE = Proportion of Variance unexplained, INT= Intrusion, AVO = Avoidance, HYP = Hyperarousal, ICC =Interclass Correlation

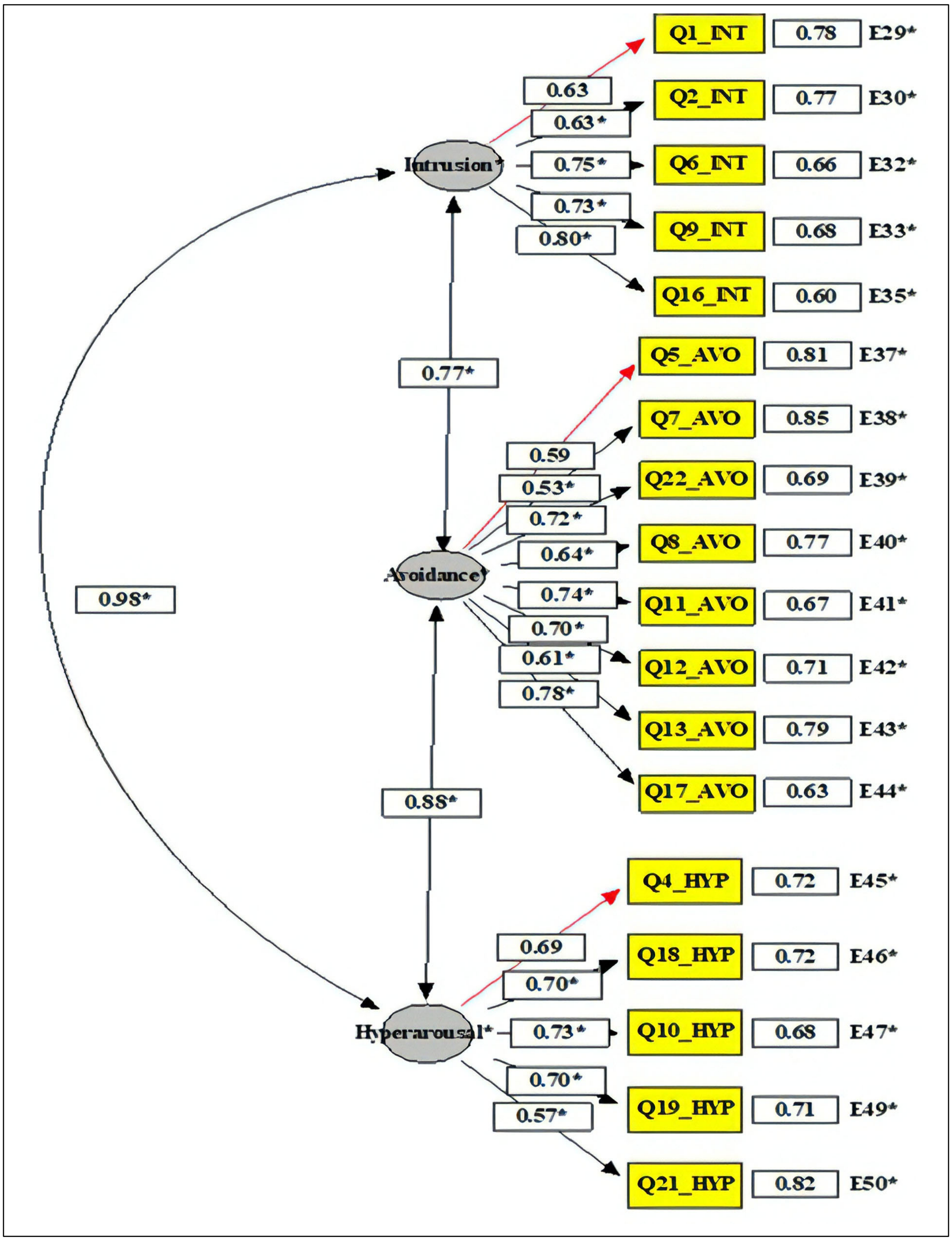

Figure S1. Confirmatory Factor Analysis for the Impact of Events Scale-Revised (IES-R).
INT= Intrusion, AVO = Avoidance, HYP = Hyperarousal
